# An Overview of the Beneficial Role of Antioxidants in the Treatment of Nanoparticle-Induced Toxicities

**DOI:** 10.1155/2021/7244677

**Published:** 2021-11-15

**Authors:** Vladimir Mihailovic, Jelena S. Katanic Stankovic, Dragica Selakovic, Gvozden Rosic

**Affiliations:** ^1^University of Kragujevac, Faculty of Science, Department of Chemistry, Radoja Domanovica 12, 34000 Kragujevac, Serbia; ^2^University of Kragujevac, Institute for Information Technologies Kragujevac, Department of Science, Jovana Cvijica bb, 34000 Kragujevac, Serbia; ^3^University of Kragujevac, Faculty of Medical Sciences, Department of Physiology, Svetozara Markovica 69, 34000 Kragujevac, Serbia

## Abstract

Nanoparticles (NPs) are used in many products and materials for humans such as electronics, in medicine for drug delivery, as biosensors, in biotechnology, and in agriculture, as ingredients in cosmetics and food supplements. Besides that, NPs may display potentially hazardous properties on human health and the environment as a consequence of their abundant use in life nowadays. Hence, there is increased interest of researchers to provide possible therapeutic agents or dietary supplements for the amelioration of NP-induced toxicity. This review summarizes the new findings in the research of the use of antioxidants as supplements for the prevention and alleviation of harmful effects caused by exposure of organisms to NPs. Also, mechanisms involved in the formation of NP-induced oxidative stress and protective mechanisms using different antioxidant substances have also been elaborated. This review also highlights the potential of naturally occurring antioxidants for the enhancement of the antioxidant defense systems in the prevention and mitigation of organism damage caused by NP-induced oxidative stress. Based on the presented results of the most recent studies, it may be concluded that the role of antioxidants in the prevention and treatment of nanoparticle-induced toxicity is unimpeachable. This is particularly important in terms of oxidative stress suppression.

## 1. Introduction

The “nano era” has emerged latterly in many different fields of science and industry. Nanotechnology refers to the development and use of small nanometer-sized objects based on their various properties. The European Commission defines nanomaterials (NM) as “Natural, incidental and manufactured materials that contain particles of which 50% or more have one or more external dimensions in the size range 1–100 nm and/or their volume-specific surface area is larger than 60 m^2^/cm^3^” [1]. There are three main classes of nanomaterials (NPs): nanoparticles, nanofibers, and nanoplates [2]. All of them have valuable and diverse use, e.g., in the electronics industry, in medicine for drug delivery, as biosensors, in biotechnology, and in agriculture, as ingredients in cosmetics and food supplements [3, 4]. They are used in paints, fillers, and filters for water purification, as catalysts, semiconductors, and opacifiers. Besides that, many nanomaterials find their purpose in material science, for making clothes, as well as in aerospace engineering [5–7]. The distinctive physical, chemical, and optical properties of nanomaterials enabled their use for a variety of purposes, but the most prominent is the progressive application in the field of medicine.

Because of their particularly interesting and unique properties, like solubility, specific surface area, aggregation state, conductivity, and high tensile strength, the metal-based nanoparticles (NP) and carbon nanotubes (CNT) have gained most of the attention of science and industry [3]. The fields of immense interest in different types of nanomaterials, e.g., lipid- or polymer-based NM, metal, metal oxide, or carbon-based NM, are medicinal and biological sciences. Regarding their small size and potential to enter the body easily, NPs have been used as drug delivery systems where they are capable to reach targeted organs or sites by cellular pathways [5]; thus, they are also used in cancer therapy, bioimaging, and diagnostics [4, 6].

As a consequence of the abundant use of nanomaterials in life nowadays, a new question has arisen concerning their potentially hazardous nature on human health and the environment in general [2, 3, 6]. In their interaction with cell membranes, many key signaling pathways may be disrupted [8]. Numerous nanotoxicological studies reported that autophagy, the main cellular process in the human organism, is affected by NMs. The disruption in autophagy can lead to many ailments such as cancer and neurodegenerative diseases [6]. By entering the cell, NPs cause the excessive formation of reactive oxygen species (ROS) which can lead to oxidative stress. The process of oxidative stress lies in the background of NP-induced cell damage and destruction, cytotoxicity, and genotoxicity [3, 9].

In recent years, there are several review papers regarding the nanoparticle-induced toxicities and their harmful mechanism of action [6, 7, 10–13] but also the positive effects of NPs synthesized using antioxidant compounds such as vitamins, minerals, natural compounds, or plant extracts [14–16]. Nevertheless, there are no comprehensive studies about the effects of antioxidants on the prevention and mitigation of severe toxicities induced by the application of nanoparticles in therapies and everyday life. In that sense, in this review, our focus was to present the recent knowledge in the field of indicative application of antioxidants to combat the deleterious effects of nanoparticles in living organisms.

## 2. The Mechanism of Nanoparticle Toxicity

Nanoparticles may enter the human body via three main pathways. The most common cause is the NP entrance by inhalation, then via the skin, and last and the most infrequent though the digestion process that depends mostly on their physicochemical characteristics. These include their hydrophilic and hydrophobic properties, particle size, shape, surface charge, and dispersity. The inhalation of NPs will transfer them into the lungs and respiratory tract, and as a result of the lower size of particles, there is an increasing concern of NPs to get deeper into the respiratory system quickly. In the dermal system, the nanoparticles will penetrate through the process of absorption but only if the skin is deeply damaged or the size of particles is below 5 nm. Ingestion of NPs rarely happens [17, 18]. After entering the body, NPs can be transferred via the bloodstream throughout the body and then accumulate and interact with various systems affecting many vital organs, such as the lungs, liver, kidneys, and reproductive organs [5, 18–21]. Additionally, the NPs can be transferred into the brain by affecting the cells and disrupting the blood-brain barrier. They can cause severe neurotoxicity; nevertheless, the way they pass through the membrane is still not sufficiently elucidated [8, 18, 22–25].

The interactions of NPs with cells lead to the disruption of many cell barriers, NPs entering the cell and causing mitochondrial damage, affecting DNA via DNA methylation and histone modifications, the development of the state of oxidative stress, and aftermost cell apoptosis. The high levels of reactive oxygen species (ROS) produced in the cell generally introduce the cell into the state of oxidative stress where proteins, DNA, and lipid structures are damaged, leading to the malfunction of the cells and severe toxicity. The oxidative stress is usually accompanied by increased expression of proinflammatory genes and activation of neutrophils and macrophages [17, 26, 27]. Nanoparticles may produce various concentrations of ROS depending on their physicochemical properties. The main properties of NPs that cause increased production of ROS are the presence of prooxidant functional groups on the NP surface, particle-cell interactions, and the existence of active redox cycling on the surface of NPs (in transition metal-based NPs) [3]. Nevertheless, the claims that oxidative stress is the most prominent factor in NP-induced toxicity have not been proven in all cases since various NPs, which have an inactive surface or low solubility, may induce toxicity without causing oxidative stress [2].

### 2.1. Nanoparticle-Induced Oxidative Stress

In most circumstances, the excess production of ROS caused by the interaction with nanoparticles underlays the formation of oxidative stress [27–29]. Oxidative stress, by its definition, represents “an imbalance between ROS production and their elimination in reaction with antioxidant defensive systems” [30]. This imbalance in the prooxidant/antioxidant relation may induce severe damage of various biomolecules like proteins, lipids, and nucleic acids, thereby causing damage to the cells and the whole organism. Although the synthesis of ROS in the organism during mitochondrial respiration or phagocytosis is a normal process, the excess in their production can be caused by various elements. If antioxidant defense systems of the organism, containing catalase (CAT), superoxide dismutase (SOD), glutathione (GSH), etc., are not capable to neutralize the increased concentration of ROS, this condition may lead to the development of severe diseases [31].

The physicochemical properties of nanoparticles significantly affect their interaction with the cell. The entrance of NPs into the cell can occur via diffusion and endocytosis or interacting with phospholipids in the cell membrane. In the physicochemical interaction with the cell membrane surface, NPs can disrupt the membrane affecting the transport mechanisms as well as induce oxidative stress by generating ions. NPs can also affect the function of cell organelles, primarily mitochondria and peroxisome, influencing the intracellular transport and therefore inducing oxidative stress [2, 18].

**Figure 1:**
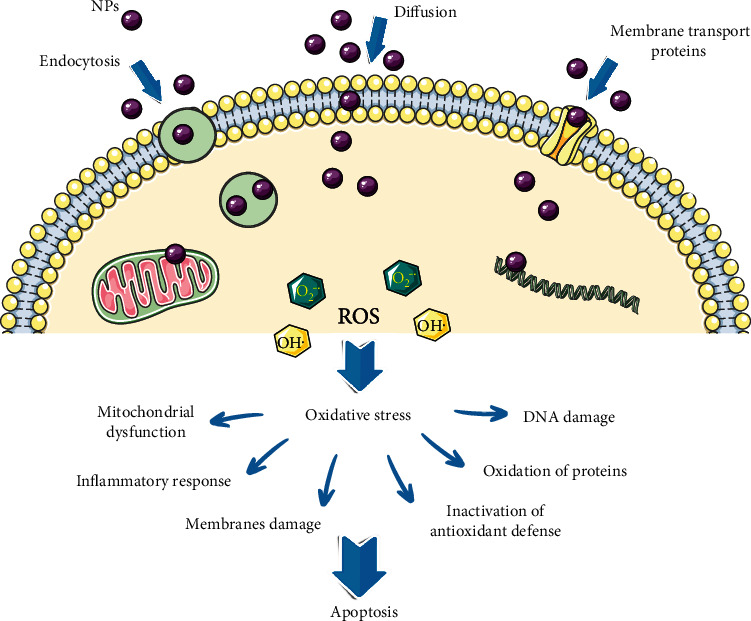
Nanoparticle-induced oxidative stress in the cell—an outline of main events.

In general, there are two types of NP-induced oxidative stress: (i) primary or direct and (ii) secondary or indirect oxidative stress (Figure 1). The first one refers to a direct reaction of the NP surface with cells inducing ROS generation. Metal-based NPs are able to release the metal ion into the cells that may also trigger an increase in ROS formation. The secondary oxidative stress may arise via indirect pathways, mainly due to NPS-induced disruption in mitochondrial function or the inability of the antioxidant defense to reinstate the redox balance. In this case, NPs are not directly responsible for the oxidative stress but affect mitochondria and phagocytes, indirectly increasing the ROS level in the cell. For instance, NPs are interacting with phagocytes (macrophages and neutrophils) whose goal is to digest them, but since NPs have often an inorganic part, the phagocytes become damaged due to their inability to neutralize the inorganic molecules. This ultimately results in an increase of the ROS level in the cell and therefore the generation of oxidative stress. NPs may affect the levels of inflammatory factors like TNF-*α* and interleukins causing mitochondrial disruption and thereafter ER stress and DNA damage. All this can finally induce the activation of apoptotic response and cell death [2, 18]. When the level of oxidative stress surpasses the ability of the organism to neutralize it, many severe conditions may occur, like inflammation, fibrosis, genotoxicity, and cancer formation [3].

The cell disturbance caused by NPs was characterized by the direct destruction of cell and organelle membranes, as well as binding to biomacromolecules with an impact on their structure and function. In addition, the NP-induced intracellular generation of ROS, also, modulates the structure and function of lipids, DNA, proteins, and carbohydrates, as main cell constituents, leading to cellular organelles and membrane damage. NP-induced toxicity involves complex mechanisms with the important role of mitochondria, lysosomes, and endoplasmic reticulum (ER) in that process [32, 33]. The intensive ROS generation has also a role in several signal pathways causing cell apoptosis, inflammation, and autophagy process. The main consequences of these processes are mitochondrial dysfunction, lysosomal, and ER damage [32]. It has been shown that the increased ROS production provoked by exposure to NPs, as well as some toxic xenobiotics, leads to mitochondrial respiration disturbance and damage of mitochondrial membrane phospholipid bilayer. The lower adenosine triphosphate (ATP) production and increased mitochondrial membrane permeability initiate apoptotic cascade and cell death [34–36]. It was also shown that the toxic concentration of different NPs accompanied by oxidative stress may disrupt the structure of the lysosomal membrane. The liberation of the lysosomal inner content to the cytosol, due to its membrane damage, could induce further damage of other organelles (especially the mitochondrial outer membrane) and further activate apoptosis [34, 37, 38]. In this regard, the use of antioxidants regulating ROS production seems a promising therapeutic strategy for NP-induced toxicity.

### 2.2. Toxicological Effects of Various Nanoparticles

In the global market of NPs, the alumina nanoparticles (Al_2_O_3_-NPs) are represented around 20% [17]. The purpose of their use is diverse, from application in medicine (for site-specific drug delivery), orthopedic implants, cosmetics, food industry, chemical engineering, catalysis, resistant coatings, lithium batteries, and all the way to jet and rocket fuels [22, 39, 40]. They also have been used in weapons, munitions, and explosives; in propeller shafts as surface coatings; also as scratch and abrasive-resistant coatings on sunglasses; and in the car industry [41]. However, their potential adverse effects on humans, animals, and the environment increase due to many ways of exposure. The most common modus of Al_2_O_3_-NPs entering into the organism is via inhalation, dermal exposure, food, and water. The small size of Al_2_O_3_-NPs and high reactivity allow easier penetration into the cells, transport via circulation, and thus the accumulation in multiple organs and tissues, e.g., the lungs, heart, spleen, testes, bone marrow, lymph nodes, and brain [22, 39]. They can also easily cross the blood-brain barrier and enter the CNS causing severe neurotoxicity. Al_2_O_3_-NP accumulation in different parts of the brain may generate memory dysfunction, depressive behavior, and neurodegenerative disorders such as Alzheimer's and Parkinson's diseases [22, 42]. Oxidative stress plays a key role in Al_2_O_3_-NP-induced toxicity in many organ systems. Recent findings showed that Al_2_O_3_-NPs provoke high production of ROS, the elevation of the MnSOD level, high levels of markers of oxidative damage (CAT, SOD, and GSH), activation of caspases, expression of endothelial cell adhesion molecules (VCAM-1, ICAM-1, and ELAM-1), and high levels of interleukins in serum. Based on published data, it can be concluded that they are triggering many adverse reactions causing an inflammatory response, mitochondrial dysfunction, cytotoxicity, genotoxicity, carcinogenicity, and apoptosis [22, 39, 42–44]. For instance, Park et al. [44] in their study related to the toxicity of aluminum NPs showed that their daily administration to mice for 28 days lead to the significant platelet increase; decrease in white blood cells, neutrophils, lymphocytes, and monocytes; and high accumulation of Al in the lung, brain, and thymus in the group treated with the highest dose. Besides, neurotoxicological effects have been observed leading to the formation of neurodegenerative and immunosuppressive effects. Another *in vivo* study by Shrivastava et al. [39] suggested that Al_2_O_3_-NPs induce a high level of oxidative stress followed by high ROS concentration, reduced levels of GSH, and low CAT and SOD activities, in mice during 7 days of an oral application. The hepatorenal toxicity of Al_2_O_3_-NPs and ZnO-NPs was monitored by Yousef et al. [43], showing that both NPs exerted significant toxicity but also synergistic toxicological effect on the liver and kidneys accompanied by systemic inflammation. Al_2_O_3_-NPs affected mitochondrial membrane potential, activation of caspases, and red blood cell dysfunction and increased ROS formation. Neurotoxicity and brain damage have been primary adverse effects in the Al_2_O_3_-NP *in vivo* application. Abou-Zeid et al. [22] reported that Al_2_O_3_-NPs caused disrupted levels of oxidative stress markers, such as MDA, 8-OHdG, GSH, CAT, and SOD; the expression of GST, TNF-*α*, and caspase-3 genes in the brain; and IL-1*β* and IL-6 levels in serum of treated animals, pointing to severe oxidative stress, inflammatory reactions, and neurotoxicity. Since NPs can also be transferred through the placental barrier, Zhang et al. [42] studied the effects of aluminum NP exposure to pregnant female mice that will influence the CNS development in the offspring. The concentration of Al in a newborn's hippocampus was significantly increased, and they showed stunted neurodevelopmental behaviors with high anxiety and impaired learning and memory performance. Taking into consideration that aluminum has many questionable deleterious effects [45], the concern of the scientific community regarding the further application of Al nanoparticles is justified.

Various calcium-containing nanoparticles (CaNPs), frequently used in composites, may also be the cause of developing serious conditions in the organism. CaNPs such as hydroxyapatite, mono-, di-, tri-, and tetracalcium phosphates as well as amorphous calcium phosphate were reported to provoke many adverse reactions in the organism. Accumulation of ROS, oxidative stress development, and cytotoxicity are just some of the consequences of CaNP use. They can affect the structure and function of various organs, like the liver, kidneys, and testes [46] and influence prodepressant behavior and cognitive impairment [23].

Cerium nanoparticles (CeO_2_-NPs or nanoceria) are widely used metal oxide nanoparticles. They are mostly applied as a diesel fuel additive to enhance combustion, as abrasive agents, in solar cells, sunscreens as UV absorbent, and contact lenses [20, 47]. Their biomedical and pharmacological application is based on their outstanding antioxidant properties. Since CeO_2_-NPs contain a small amount of Ce^3+^ ions, the redox reactions between the Ce^3+^ and Ce^4+^ open the possibility of nanoceria to react with free radicals like O_2_^−•^ and ^•^OH, therefore establishing a function similar to CAT and SOD. Based on these criteria, they can be used to combat oxidative stress in the organism so their yearly production of around 10 000 t is not surprising [47–49]. The CeO_2_-NP dermal and intestinal absorption is unlikely; therefore, the main route of entering the organism is by inhalation into the respiratory tract [47]. Although it could be concluded that the antioxidant effects of CeO_2_-NPs can only bring benefits, the *in vivo* studies showed that inhalation of CeO_2_-NPs can induce severe damages in the respiratory system, pulmonary tissues, and systematic toxicity. The investigation of Ma et al. [20] showed that due to the exposure of rats with CeO_2_-NPs, the NO production was reduced but IL-12 production in alveolar macrophages increased leading to the activation of caspases 3 and 9 and alveolar macrophage apoptosis. Arginase-1 and osteopontin were elevated in lung cells. CeO_2_-NPs induced significant lung inflammation and damage of tissue that may cause fibrosis [20]. Another *in vivo* research reported tissue distribution of inhaled CeO_2_-NPs in rats after a 28-day exposure [50]. Geraets et al. came up with astonishing results that nanoceria particles were distributed in every monitored tissue (lung, liver, kidney, spleen, brain, testis, and epididymis) after a single 6 h exposure. Moreover, repeated exposures lead to a significant accumulation of CeO_2_-NPs in tissues. Besides severe toxicity in the respiratory tract, hepatic, neural, and dermal toxicities of CeO_2_-NPs were also reported [47].

Titanium dioxide nanoparticles (TiO_2_-NPs) are in high use in medicine, cosmetics, and industry. They are added to sunscreen, toothpaste, food, and various paints and are also used for drug delivery and in wastewater treatment, due to their photocatalytic, UV-protective, antibacterial, and self-cleaning properties [29, 51]. TiO_2_-NPs have the ability to absorb photons after exposure to UV light, but photoexcited TiO_2_-NPs can also induce high production of ROS, thus triggering a state of oxidative stress in live organisms [52]. The rising concern regarding human exposure to TiO_2_-NPs is more than justified. There are two crystalline forms of TiO_2_, anatase, and rutile. Anatase is a frequently used form in sunscreens (regulated by the United States Food and Drug Administration); thus, dermal exposure to TiO_2_-NPs can be quite high leading to possible keratinocyte toxicity and skin allergy responses [53]. TiO_2_-NP accumulation may cause severe problems in heart function, developing oxidative stress, inflammation, and atherosclerosis. The study of Hong et al. [51] showed significant TiO_2_-NP-induced cardiac lesions and pulmonary inflammation in mice, with high levels of oxidative stress parameters. Besides the skin and hearth tissue, TiO_2_-NPs were reported to accumulate in other vital organs, like the kidney, liver, lung, spleen, and brain, leading to apoptosis and organ failure [19, 29]. One of the most serious toxicities of TiO_2_-NPs was observed in the reproductive system. Because of their physicochemical properties and small size, TiO_2_-NPs can easily go through the blood-testis barrier, accumulate and damage testes tissue, and disrupt all vital functions [54]. Gao and coworkers [19] reported that the application of a low dose of TiO_2_-NPs during a long period caused severe testicular tissue damage accompanied by sperm lesions and reduced spermatogenesis in mice. The expression of the genes included in the process of spermatogenesis was also disrupted. Many similar results should raise awareness of the TiO_2_-NP negative effects on human health [19].

The iron oxide nanoparticles (IONPs) can be of various types of oxides depending on the ferrous valence, such as magnetite (Fe_3_O_4_), hematite (*α*-Fe_2_O_3_), and maghemite (*γ*-Fe_2_O_3_). The bioavailability of IONPs is very high, and they can be located in certain tissues by the influence of an external magnetic field. In that sense, they find their application mostly in medicine (magnetite and maghemite), for various purposes like drug delivery, therapy of cancer and thermal ablation, and magnetic resonance imaging (MRI). Even FDA approved some of the IONPs, ferumoxytol and ferumoxides, for use in MRI. Since IONPs are superparamagnetic, they can be used for medical imaging or magnetic drug targeting (MDT) [55, 56]. Various studies have been reporting the discrepant results on IONP toxicological effects, some claiming that there is no significant toxicity while others reported severe consequences. A recent study dealt with *in vivo* toxicity induced by ultrafine IONPs in rats [56]. The results of 4 weeks of exposure to IONPs showed to be decreasing in bone marrow-mononuclear cell proliferation, with high ROS levels, increased inflammatory response, and DNA changes leading to an apoptotic outcome. Although structural spleen tissue damage had not been noticed, the level of oxidative stress markers in tissue was extremely high, suggesting that high doses of IONPs may cause significant toxicity in the organism [56, 57]. One can be exposed to IONPs also via inhalation, and by entering the respiratory tract, these nanoparticles may become extremely deleterious, thereby causing pulmonary inflammation, tissue fibrosis, changes in pulmonary function, and immunological response. Zhang and coworkers [58] showed that the treatment with Fe_3_O_4_-NPs can induce high toxicity in the human bronchial epithelial cells by cumulative oxidative stress, whereby low GST, SOD, and CAT activities were detected.

The application of copper oxide nanoparticles (CuO-NPs) is quite versatile, from industrial use as additives in inks, medical devices, and metallic coatings, up to medicinal purposes due to their antibacterial, antifungal, and anti-inflammatory properties [59, 60]. Although the use of CuO-NPs in nanomedicine showed many benefits, for drug delivery, as a contrast agent, and in diagnostics, their overaccumulation in the human body may lead to pronounced consequences, mainly via inducing oxidative stress [61]. Like the abovementioned NPs, because of their size, CuO-NPs can easily cross biological barriers, therefore reacting with biomolecules, inducing ROS synthesis and accumulation, which further evokes oxidative stress and damage on various levels [60]. They interact with biological membranes, DNA, and proteins, causing severe damage and inactivation, liver and kidney toxicities, brain dysfunction, and metabolic alkalosis [62].

One of the most important and the most used nanoparticles is zinc oxide nanoparticles (ZnO-NPs). ZnO-NPs have been listed as safe substances by the US FDA so that their use increased sharply in recent years [63]. They can be synthesized by various methods and used in different fields, such as the rubber, textile, electronics, electrotechnology, and food packaging industries, in concrete production, in photocatalysis, and as pigments and coatings. ZnO-NPs are quite used in the cosmetic industry, in sunscreens, based on their valuable UV absorption effects, but also in many other products because of their remarkable antimicrobial properties [63, 64]. Although these NPs are generally considered to be safe, some aspects of their potential to induce toxicity should be mentioned. ZnO-NPs can induce various toxicities accumulating in the human organism, but the exact mechanisms of their toxicity are still quite unknown [28, 65]. Pandurangan and Kim [64] explained the most likely mechanisms of ZnO-NP action in the cells causing severe damage based on their high solubility. One is that the high extracellular concentration of these NPs may lead to an increase of the Zn^2+^ level inside the cells cutting down the activity of the Zn-dependent enzymes and transcription factors. Another mechanism of ZnO-NP toxicity can arise when they enter the cell where they can affect the structure of enzymes and transcription factors, and the last mechanism is via disrupting the pH level caused by dissolution of ZnO-NPs in the lysosomes. Cytotoxicity of ZnO-NPs was demonstrated in a study designed by Yu et al. [28] where normal skin cells were exposed to ZnO-NPs. It was shown that ZnO-NPs induced the formation of ROS in high concentrations, leading to oxidative stress development, autophagic vacuole accumulation, and mitochondria dysfunction. Cytotoxic effects and genotoxicity of ZnO-NPs were also demonstrated on human SHSY5Y neuronal cells [24]. Although zinc NPs did not enter the neuronal cells, they caused cell death via various damages of the cell cycle, DNA, and cell structure. *In vivo* studies on ZnO-NPs reported similar findings and the possibility of developing serious disorders. ZnO-NPs, at concentrations of 200 or 400 mg/kg/day (for 90 days), induced a state of high oxidative stress in mice [65]. The level of liver injury was enormous, including tissue disruption, reduced concentration of GSH, high levels of transaminases in serum, and endoplasmic reticulum stress which lead to apoptosis. Similar results were obtained by Yousef et al. [43] in the study on male Wistar rats treated, not just with ZnO-NPs, but also with Al_2_O_3_-NPs. It was shown that the oral administration of those NPs, alone and together, induced high toxicity in the liver and kidneys with the loss of function, oxidative stress, tissue damage, and systemic inflammation, with highly synergistic action.

Gold nanoparticles (AuNPs) are recognized and FDA approved for their biomedical application, drug delivery, biosensing, cell imaging, gene therapy, and radiotherapy but also find use in the food industry, water purification, and alleviation of pollution [13]. Nevertheless, certain studies revealed potential harmful effects of AuNPs on humans and the environment. After entering the organism, via previously mentioned routes, AuNPs can induce inflammation and cytotoxicity, increasing levels of oxidative stress. Abdelhalim et al. [66] conducted an *in vivo* study in which male Wistar-Kyoto rats were intraperitoneally treated with AuNPs for 7 days. AuNPs significantly elevated the oxidative stress markers, but also the parameters of liver function, causing hepatotoxicity. Gold NPs also may affect the red blood cells (RBCs), causing hemoglobin deoxygenation [4]. The same study reported similar activity of silver nanoparticles (AgNPs) on RBCs, additionally producing ROS and therefore high oxidative stress levels and cell damage. The conclusion was derived that changes in the structure of hemoglobin were mainly due to pH shifting in the cytoplasm [4].

Silver nanosized particles are used, to the greatest extent, for their immense antimicrobial properties, like silver itself. AgNPs proved their effects as antibacterial, antifungal, and antiviral agents [17] so their usage in biomedical purposes relies on these properties. Since they are FDA approved for antibactericidal purposes, over four hundred products on the market contain these NPs [67]. They are applied in wound dressings, but also as the coating of medical appliances, like surgical instruments or prosthetics [5]. AgNPs have also been applied, as many previously mentioned NPs, for drug delivery, molecular imaging, and even cancer therapy, but in the food and textile industry too [10, 17]. Due to the widespread use of AgNPs, there is, again, reasonable concern whether these NPs can harm live organisms. The route of uptake of AgNPs does not differ much from the above stated. In the cells, AgNPs can accumulate and release Ag^+^ ions, therefore affecting the cell function by provoking oxidative stress, damage of the mitochondria and genetic material, and, ultimately, apoptosis [3, 10, 67]. They are also able to readily transfer the blood-brain barrier, reach the brain tissue, and provoke severe consequences [17]. Thus, AgNP-induced neurotoxicity was investigated by Yin et al. [25] on neonatal Sprague-Dawley rats and it was shown that AgNPs induced significant alterations in neuronal tissue. Hepatic tissue can also be affected by AgNPs [5], where they induce high levels of oxidative stress (observed through CAT, SOD, MDA, and GSH levels) and increase serum markers of liver function (transaminases, alkaline phosphatase, and proteins), accompanied with tissue changes and DNA damage. Besides the accumulation in organs, AgNPs have extensive toxicity on the human sperm. Wang et al. [27] reported a dose- and time-dependent change in sperm viability and motility after treatment with AgNPs with high levels of ROS and DNA damage. Treatment of freshwater snail (*Lymnaea luteola* L.) with silver NPs lowered the levels of GSH, glutathione-S-transferase, and glutathione peroxidase while lipid peroxidation was significantly elevated as well as DNA damage in digestive gland cells [68].

## 3. Antioxidants

The antioxidants can be defined in different ways, but one of the most simple definitions is that they are molecules able to protect the various section of biological systems against oxidative damage [69]. They are able to act in the prevention of the damage, to scavenge and neutralize free radicals and reactive oxygen and nitrogen species, and to repair new antioxidants, thus counteracting their action, inhibiting the oxidation of biologically important molecules [70]. In that sense, antioxidants possess an important role in aerobic living organisms. Essentially, this group of different components in organisms possesses a high ability to prevent oxidative stress (preventing reactions of free radicals with biomolecules), terminate radical oxidation reactions, and repair the damage induced by free radical reactions [71]. The intensive production of free radical and reactive species in humans leads to an imbalance between the rate of their formation and the antioxidant defense of the organism leading to pathological processes called “oxidative stress.” This imbalance may be provoked by intense exposure of the organism to exogenous harmful factors such as UV and radioactive irradiation, pollutants, xenobiotics, smoking, heavy metals, and extreme physical exertion. This may be a cause of different tissue and organ damage, as well as different disease promotions [72]. The exogenous nonenzymatic antioxidants such as mineral elements, vitamins, dietary supplements, or plant antioxidants represent an important source of compounds for support of the human antioxidant defense system in the prevention and mitigation of organism damage caused by oxidative stress [70, 73].

The most used antioxidants among the human population are vitamins, such as vitamin A, vitamin C, and vitamin E and, then, *β*-carotene, minerals (like Se), and plant polyphenols. Regardless of their importance for human halt and vitality, they can cause adverse effects if consumed in much higher doses than those found in foodstuffs. Scientists have reported that long-term consumption of high dosages of antioxidant supplements (vitamins A, C, and E and *β*-carotene) may be associated with an increased risk of some disorders in humans. Researchers reported that the most beneficial use of antioxidant supplements may be in the case of their deficit for normalization of their levels [74, 75].

Despite the high efficacy and a high number of currently known natural or synthetic antioxidants, there are some limitations in their specific applications in biomedicine, food industry, pharmaceutical, and cosmetic products. Sometimes, the main problem for their application is possible toxic effects, self-retention in the desired location, and sensitivity to atmospheric oxygen or enzyme degradation. Antioxidants in the form of nanoparticles have been recently proposed as an innovative solution for the improvement of their characteristics. Advancement in nanotechnology has revealed several nanoparticles consisting of biologically originated molecules with antioxidant activities, such as lignin, melanin, coenzyme Q10, or polyphenol nanoparticles [38, 76, 77]. Many antioxidant compounds are developed as nanoparticles functionalized with antioxidants. This type of nanoparticle antioxidants may possess a core with a surface consisting of covalently bound antioxidants (magnetic nanoantioxidants) or nanoparticles as passive carriers able to deliver and release antioxidants (e.g., nanoencapsulated, nanotubes, or mesoporous materials). There is a number of functionalized nanoparticles, e.g., Fe_3_O_4_ or graphite-coated cobalt magnetic NPs functionalized with different natural or synthetic antioxidants, as well as nanoencapsulated antioxidants [76, 78, 79].

Considering the wide use of different NPs in many products and materials for human use, as well as due to the people exposure risk workplaces and their existence in the environment [29, 80], there is increased interest of researchers to provide possible therapeutic agents or dietary supplements for the amelioration of nanoparticle-induced toxicity. In this context, the authors focused in this review, to summarize the knowledge about the use of antioxidants as supplements for prevention and alleviation of harmful effects caused by exposure of organisms to NPs. The studies in this field were searched using Scopus, Google Scholar, Science Direct, and PubMed. The most relevant publications were selected based on the following keywords: “nanoparticle-induced toxicity,” “prevention of nanoparticle-induced toxicity,” “effects of antioxidants on nanoparticle-induced toxicity,” “antioxidants and nanoparticles,” “plants and nanoparticle-induced toxicity,” “plant extracts and nanoparticle-induced toxicity,” and “bioactive compounds in nanoparticle-induced toxicity.” The references from 2010 until 2021 are included in this review.

### 3.1. Vitamins and Dietary Supplements

Vitamin E (*α*-tocopherol) is one of the most important carotenoids with remarkable antioxidant properties. It is able to neutralize ROS and decrease the lipid peroxidation reactions in the organism [81]. Therefore, the idea of its application to counteract the NP-induced oxidative stress is not surprising. Most of the recent *in vivo* studies used silver nanoparticles for inducing toxicity. For example, Hedayati et al. [82] used the zebrafish (*Danio rerio*) model for the evaluation of vitamin E protective effects towards AgNP-induced toxicity. Vitamin E was applied as a food supplement in three different doses. The results showed that AgNPs induced significant immunological impairments with inhibition of lysozyme and ACH50 (alternative complement pathway) activity, cellular damage with increased LDH activity and cortisol levels, and high levels of oxidative and metabolic stress by lowering of inhibiting CAT and SOD activities. Higher doses of vitamin E were able to significantly protect the organism from AgNP action, restoring all vital parameters [82]. The lipophilic nature of vitamin E grants its use as a neuroprotective agent, but the studies of its effect against neurological impairment induced by NPs are rare. One of these is the study of Yin et al. [25] dealing with the AgNP-induced neurological toxicity in neonatal Sprague Dawley rats. A series of deleterious neurotoxic effects of nasal administration of AgNPs were reported, including structural disorders in the cerebellum, stress, and body weight loss. Vitamin E oral supplementation exerted strong neuroprotective effects and was able to improve the bodyweight of animals and reduce the level of astrocyte activation or proliferation, but it was unable to significantly ameliorate AgNP-induced neurohistological changes [25]. Recently, another *in vivo* study showed valuable effects of vitamin E on AgNP-induced degeneration of filiform and circumvallate tongue papillae [83]. The albino rats were exposed to AgNPs and vitamin E for 28 days. The immunohistochemical and histological examinations showed valuable protective effects of vitamin E administration in terms of protecting both tongue papillae of AgNP toxic effects and apoptotic changes. The combination of lipophilic vitamin E and hydrophilic vitamin C proved to be efficient against toxicity induced by zinc oxide nanoparticles (ZnO-NPs) in fish species Nile tilapia (*Oreochromis niloticus*) [84]. The oxidative stress parameters, such as glutathione reductase (GR), glutathione peroxidase (GPx), and glutathione-S-transferase (GST) activities and gene expression, the levels of glutathione (GSH) and lipid peroxidation, in the liver and gill of Nile tilapia were monitored. It was shown that ZnO-NPs significantly altered all parameters, but the mixture of vitamins E and C was able to reduce the levels of oxidative stress in Nile tilapia by upgrading all parameters within normal limits. The synergistic effects of tocopherols with vitamin C, where vitamin C is able to regenerate tocopherol activity, seem to be a crucial factor for their use as a mixture [72].

Another lipophilic vitamin used as an antioxidant is vitamin A (retinol) which showed significant activity in different cellular processes, and it is essential for the vision and reproductive system. Its activity, in mixture with vitamin E, regarding TiO_2_-NP-induced toxicity was monitored by several recent studies. Khanvirdiloo et al. [85] evaluated testicular changes induced by titanium dioxide nanoparticles (TiO_2_-NPs) and how vitamin A, vitamin E, and their combination can alter those changes in male Wistar rats. TiO_2_-NPs caused severe damage to the spermatogenesis process; it decreased sperm count, motility, and viability, sperm chromatin integrity was disturbed, and inflammation in testicular tissue was observed. Nevertheless, the administration of vitamins A and E, particularly their mixture, had profound effects on reducing the testicular toxicity of TiO_2_-NPs. Besides testicles, TiO_2_-NPs can be accumulated in many other organs where they may provoke severe implications. The spleen is very susceptible to TiO_2_-NP accumulation and deleterious action. Afshari-Kaveh et al. [86] reported severe changes in the oxidative status of spleen tissue of Wistar rats treated with TiO_2_-NPs. Nanoparticles induced significantly increased total oxidant status and lipid peroxidation levels. The total antioxidant capacity in spleen tissue was decreased likewise SOD and GPx activities and their gene expression. Nevertheless, the treatment with vitamins A and E, separately and as a mixture, showed outstanding antioxidant properties in terms of reinstating the levels of antioxidant parameters back to normal as well as protecting spleen tissue from histological changes induced by TiO_2_-NPs.

Vitamin D, also called “the sunshine vitamin,” has a crucial role in promoting bone health in children and adults as well as lowering the potential formation of chronic diseases, including cancer and cardiovascular disorders. It serves as a membrane antioxidant but also as a regulator of endogenous antioxidant defense systems. Generally, vitamin D exists in its inactive form, whether made in the skin or ingested, but becomes activated by hydroxylation in the liver and kidneys [87]. Its protective role towards the liver and kidneys was studied in the state of oxidative stress induced by manganese oxide-nanoparticles (MnO_2_-NPs) [88]. Although MnO_2_-NPs can affect environmental conditions, their possibility of entering into the human organism, via previously mentioned routes, is of great concern. They can be toxic on different levels, wherever they accumulate, including the possibility to penetrate the blood-brain barrier. In the BALB c mice, significant toxicity was developed after exposing them to MnO_2_-NPs and the levels of liver and kidney functions were substantially lowered while serum bilirubin and glucose concentrations were much higher compared with the control group. The intraperitoneal administration of vitamin D for 50 consecutive days showed improvement in liver and kidney functions with a reduction of disrupted serum parameters. Taking that into account, vitamin D exerted significant hepato- and nephroprotective effects against MnO_2_-NP-induced toxicity [88].

Hydrophilic vitamin C (L-ascorbic acid) is known as a very potent antioxidant compound. Since it is easily soluble in water, it can react directly with free radicals or its action may be indirect via reinstating the antioxidant activity of liposoluble vitamin E, as mentioned previously [89]. Besides its activity as a radical scavenger, it may also react as a chelator of heavy metals. Vitamin C has many beneficial effects on human health, it prevents heart disease; improves the function of cartilage, joints, and skin; has profound effects on the immune system; increases nutrition absorption; and has antigenotoxic and anticarcinogenic potential [90]. In *in vitro* assays, in human lung carcinoma, A549 cells showed significant toxicity of ZnO-NPs. Exposure to vitamin C leads to a decrease in intracellular ROS production which lowered the inflammation level [91]. The proposed mechanism of vitamin C action was based on its antioxidant activity and chelating reaction with Zn leading to the formation of a stable complex. The *in vivo* fish model, common carp (*Cyprinus carpio*), was used for the evaluation of vitamin C protective activity against TiO_2_-NP-induced toxicity [92]. It was reported that TiO_2_-NPs significantly increased the level of oxidative stress in the organism, which can be seen through increased levels of glucose and cortisol; higher activity of ALT, AST, and ALP; and decreased immune parameters. Liver tissue damage was also observed in the group treated only with TiO_2_-NPs. Supplementation with vitamin C, at a concentration of 500 to 1000 mg/kg of feed, decreased the level of tissue damage and mainly restored oxidative stress parameters preventing severe consequences which may have arisen due to exposure with TiO_2_-NPs. Vitamin C has also proven itself in the protection of rats against reproductive toxicities and oxidative stress induced by nickel nanoparticles (NiNPs) as reported by Kong et al. [93]. NiNPs induced severe consequences in rats' testicular tissue function; the levels of CAT, SOD, and gonad-stimulating hormone (GSH) were disrupted, with increased levels of ROS, nitric oxide, and lipid peroxidation. Also, NiNPs affected caspases 9, 8, and 3 and expression of Bcl-2-associated X protein (Bax) and apoptosis-inducing factor (AIF). Vitamin C upregulated all parameters and ameliorated NiNP-induced reproductive toxicity mostly due to its antioxidant properties [93].

Besides vitamins, many other compounds used as dietary supplements can serve as natural antioxidant supplementation to help combat NP-induced oxidative stress. One of those is selenium (Se), an essential trace element with valuable antioxidant and anticancer activities [94]. In the *in vivo* study by Ansar et al. [95], sodium selenite was used in the treatment of rats exposed to silver nanoparticles (AgNPs). AgNPs applied at a concentration of 5 mg/kg/b.w. induced substantial oxidative stress in animal testes, by reducing GSH levels, GPx, SOD, and CAT activities and, on the other hand, increasing the levels of lipid peroxidation and expression of interleukins (IL-1*β* and IL-6) and tumor necrosis factor-alpha (TNF-*α*). Moreover, the testes tissue damage induced by AgNPs was prominent and spermatogenesis was affected. Sodium selenite (0.2 mg/kg/b.w.) was able to improve all parameters of oxidative stress defense and inflammatory markers, including testicular tissue morphology. Although the exact mechanism of Se antioxidant effects against AgNP-induced toxicity is not known, its beneficial role in reestablishing endogenous antioxidant defense mechanisms should be acknowledged.

A sulfur-containing amino acid N-acetylcysteine (NAC) is known as an impressive free radical scavenger and antioxidant. It serves as a contributor to L-cysteine, in relation to which it has a more stable structure, as a precursor in glutathione synthesis, thus regulating the intracellular levels of GSH. Antioxidant effects of NAC are realized through releasing of sulfhydryl groups to reduce ROS levels. NAC can react with various free radicals such as hydrogen peroxide, superoxide, and peroxynitrite. It can also act on the reduction of the NF-*κ*B pathway and secretion of inflammatory cytokines. In the state of oxidative stress, NAC plays a crucial role in preventing and reducing the damage that may arise [96, 97]. The effects of NAC on cobalt nanoparticle- (CoNP-) induced cytotoxicity in a mouse renal tubular epithelial cell model (TCMK-1 cell line) were monitored *in vitro* [98]. The application of CoNPs induced a higher rate of cell apoptosis; increased the p-ERK, p-p38, and p-JNK expression; and activated the MAPK pathway. NAC was able to reverse the cell death process and inhibited ROS-induced p-ERK, p-p38, and p-JNK MAPK pathways. These findings support the fact that NAC has an exceptional antioxidant potential which can find its application in NP-induced oxidative stress [98]. In that sense, an *in vivo* study conducted in male albino rats used titanium dioxide nanoparticles (TiO_2_-NPs) for inducing the testicular toxicity [97]. As expected, TiO_2_-NPs caused severe histological changes in testes tissue accompanied by positive TNF-*α* immunoreaction and DNA damage. Lipid peroxidation in serum was highly elevated while GSH and testosterone levels were reduced. The treatment with NAC had an impact on all parameters and lead to their restoration, with minor antigenotoxic effects. Therefore, its antioxidant potential was significantly expressed in this state of TiO_2_-NP-induced oxidative stress.

Another amino acid, L-arginine (Arg), defined as a conditionally essential amino acid, can be implemented in the treatment of NP-induced toxicities. Recently, Abdelhalim et al. [66] conducted an *in vivo* study using rats as a model organism. They were treated with gold nanoparticles (AuNPs), and the level of oxidative stress was monitored via estimation of crucial markers (ALP, ALT, GGT, total protein, MDA, and GSH). The AuNP administration induced significant hepatotoxicity and increased oxidative stress levels. The use of arginine proved to be very successful in terms of alleviation of all oxidative stress parameters thus acting protectively against the influence of AuNPs.

*α*-Lipoic acid, also known as thioctic acid, is a naturally occurring organosulfur compound that can be synthesized by plants, animals, and humans. It is often used as a dietary supplement due to its remarkable bioactive properties, particularly the antioxidant potential. *α*-Lipoic acid can act as a direct antioxidant by scavenging reactive oxygen and nitrogen species, or it may activate various antioxidants and regulate other signaling pathways [99]. This supplement has been used as additional therapy in the state of mesoporous silica nanoparticle- (MSiNP-) induced oxidative stress [100]. Primarily, Sun et al. designed *in vitro* experiment on the human neuroblastoma SH-SY5Y cell line which showed that MSiNPs were able to inhibit cellular proliferation via ROS generation that further entails impaired mitochondrial function and apoptosis activation. The *in vivo* part of the study was conducted on mice and showed high levels of oxidative stress and disrupted the brain function due to the easy transition of MSiNPs through the blood-brain barrier. *α*-Lipoic acid was used for modification of MSiNPs and showed a reduction in the oxidative stress level, alleviation of the cytotoxicity both *in vitro* and *in vivo*, and reduction of NP toxicity due to its significant antioxidant effects [100]. The combination of *α*-lipoic acid and vitamin E turned out to be great in the treatment of AuNP-induced nephrotoxicity in rats [101]. Since AuNPs caused severe changes in renal tissue and, again, high levels of oxidative stress, *α*-lipoic acid and vitamin E were able to reduce lipid peroxidation, inflammation, and toxicity by increasing antioxidant defense in the organism.

### 3.2. Plant-Based Antioxidants

The use of medicinal and edible plants is intensively studied in the prevention and treatment of oxidative stress ailments [73, 102, 103]. The potential use of plant extracts as antioxidant supplements for the mitigation of nanoparticle-induced toxicity has intensively been researched in recent years.

The most extensive research about the ameliorated effects of plant extracts or essential oils has been conducted on TiO_2_-NP- and AgNP-induced oxidative stress using different model organisms (Table 1). The use of *Tinospora cordifolia* ethanol extract in experiments with Nile tilapia (*Oreochromis niloticus*) fish showed that a standard fish diet supplemented with this plan extracts can regulate antioxidant parameters in fish gill, liver, and kidney, as well as inflammation in the liver induced by TiO_2_-NPs [104]. *Rosmarinus officinalis* is reported as a plant that successfully ameliorated plasma antioxidant markers (CAT, SOD, MDA, and total antioxidant status (TAS)), the IL-6 level, and DNA damage in rats treated with TiO_2_-NPs [105]. The modulation of hepatotoxicity induced by TiO_2_-NPs in rats showed grape seed standardized (based on proanthocyanidins (95%)) extract [106] and cinnamon bark extract [107] enhancing oxidative parameters in liver tissue. Besides the hepatoprotective activity of cinnamon bark extract, an encapsulated cinnamon essential oil also possesses protective properties of TiO_2_-NP-induced oxidative stress. It is observed that the treatment of mice with maltodextrin-encapsulated cinnamon essential oil significantly reduced oxidative markers in the liver and kidney caused by TiO_2_-NPs. In the same study, cinnamon essential oil reduced serum cytokines levels, DNA fragmentation in the hepatocytes, chromosomal aberrations in bone marrow cells, and sperm shape abnormalities of male mice treated with TiO_2_-NPs [108]. Abdou et al. [109] demonstrated that *Moringa oleifera* leaf extract possesses nephroprotective potential in rats treated with TiO_2_-NPs. This plant extract decreased oxidative stress in the kidneys induced by TiO_2_-NPs, as well as modulated expression of NF-*κ*B, Nrf2, and HSP-70 attributed to increased oxidative stress. Another study also showed that *M. oleifera* seed extract displayed a similar effect on TiO_2_-NP-induced cerebral oxidative damage in rats [110]. *Moringa oleifera* seed extract was also applied in the prevention of copper nanoparticle- (CuNP-) induced toxicity in *Cyprinus carpio* fish, suggesting that this extract may successfully normalized lipid peroxidation, GSH level, and CAT activity in gill and liver tissues of fish [60].

*In vitro* studies published about AgNP-induced toxicity showed that studied NPs induced ROS formation leading to DNA damage of human embryonic kidney (HEK 293) cells. Pretreatment of these cells with *G. asclepiadea* extracts showed prevention of AgNP-induced DNA damage determined in comet assay and reduction of oxidized base lesions (8-oxoG) compared with cells that were not pretreated with the extracts [114, 115]. Experiments performed on Nile tilapia fish demonstrated the beneficial role of diet supplemented with pomegranate (*Punica granatum*) peel when the fish were exposed to sublethal levels of AgNPs (up to 2.0 mg/L) for six weeks. In this study, pomegranate peel supplementation significantly improved biochemical parameters in blood related with liver and kidney function, antioxidant markers in liver and kidney tissues, and immunity biomarkers of the fish exposed to sublethal levels of AgNPs [120]. The hepatoprotective properties of several plant species were also studied *in vivo* against AgNP-induced hepatotoxicity. *Ginkgo biloba* aqueous extract showed an important influence on liver function and the antioxidative status of rats treated with AgNPs (50 mg/kg b.w.) upregulating PGC-1*α*, mtTFA, and Nrf2 mRNA mitochondrial transcription factors [116]. In another study, standardized *G. biloba* extract to 24% ginkgo flavonoids improved oxidative damage in the brain of rats treated with AgNPs, as well as significantly regulated proinflammatory cytokine gene expression in brain tissue [117]. Pomegranate [123] and beetroot [5] juices also provided significant hepatoprotective activities against AgNP-induced toxicity in animal experiments. Albrahim and Alonazi [5] showed that beetroot juice posttreatment has the potential to regulate apoptotic proteins p53 and Bcl-2 in liver tissue of rats treated with AgNPs. Pomegranate juice also displayed the decrease of the MDA level and the increase of the GSH level in the liver and kidney of rats intoxicated with CuO-NPs, regulating caspase-3, Bcl-2 levels, and NF-*κ*B disturbed expression caused by overproduction of ROS [122]. In another publication, Hassanen et al. [121] showed that pomegranate juice can reduce oxidative stress manifestations in the brain of rats treated with CuO-NPs through regulation of HO-1 and Nrf2 expression, important for cellular redox balance. Green tea extract also showed significant improvement of hepatotoxic manifestation caused by CuO-NP application in rats, improving the oxidative status of the liver and regulating the expression of the caspase-3 and Bax proteins [59].

Essential oils of *Ocimum basilicum* L. (basil) and *Zataria multiflora* Boiss. were studied for hepatoprotective activity against IONP-induced toxicity. Both essential oils had the ability to prevent hepatotoxicity of IONPs in rats regulating antioxidant parameters in liver tissue of experimental animals [111, 124]. A few published studies examined the application of plant extract or oils for the prevention of toxic effects of lesser-extent-investigated NPs. Pumpkin seed oil was applied in the study for the determination of its protective effects against Al_2_O_3_-NP-induced toxicity in pregnant rats. The oil possessed the ability to enhance antioxidant parameters in maternal and fetal hepatic and brain tissues of pregnant rats with developed Al_2_O_3_-NP-toxicity [125]. Alotaibi et al. [112] used *Eruca sativa* L. seed extract for the treatment of hydroxyapatite nanoparticle-induced toxicity concluding that this extract improved antioxidant parameters (SOD, CAT, GSH, and TBARS) in heart tissues of hydroxyapatite NP-treated rats. *Filipendula ulmaria* extract also showed a positive influence on CaNP- and hydroxyapatite NP-induced oxidative stress in brain tissue of rats [23], but also in liver, kidney, and testes tissues [46]. The essential oil of *Pistacia lentiscus* L. lowered ROS generation and stimulated SOD and CAT activities in human lung epithelial cells (A549) exposed to NiO-NPs [119]. SiO_2_-NPs-induced toxicity in rats described by El-Sayed et al. [118] was ameliorated using ginseng dried plant which could reduce oxidative stress, as well as apoptotic and inflammatory processes in rat lung. The mixture of *Foeniculum vulgare* (fennel) and *Pimpinella anisum* (anise) seed extracts showed hepatoprotective potential significantly lowering oxidative stress in liver tissue of rats exposed to ZnO-NPs [113].

In terms of reducing NP toxicity, many plant extracts are frequently used in the eco-friendly synthesis of NPs with lower toxic effects. NPs obtained in these processes usually display additional pharmacological properties compared with conventionally synthesized NPs. Besides, plants represent a renewable, environment-friendly, and widely available material for NP synthesis. This relatively new approach in NP synthesis is in the research focus in recent years [14, 156, 157]. Hence, plants are also important for the development of new methods for the synthesis of less-toxic NPs as well as for the suppression of NP toxicity. Analyzed literature data about the use of plant extracts or essential oils in the prevention and reduction of NP-induced oxidative stress showed that some plants may be utilized as effective supplements in this type of oxidative damage. Most studies deal with research about the use of aromatic and edible plants in NP-induced oxidative stress that emphasizes and encourages the consumption of these plant species in the prevention and fight against oxidative stress. The highest number of analyzed studies reported that examined plant products exert hepatorenal protection in experimental animals reporting essential results about the oxidative status of organ tissues. The significantly lower number of studies deals with a profound analysis of protection mechanisms of NP-induced oxidative stress using plants. Hence, there is a need for more research with comprehensive results about the application of plants in the suppression of NP-induced oxidative stress. All these results open the possibility for further research of plants in this field.

### 3.3. Phytochemicals

Different plant constituents are well known for their excellent antioxidant properties; among them, phenolic compounds and some components of essential oils possess the most pronounced antioxidant properties [70, 157]. In this regard, phytochemicals with high antioxidant potential are frequently used in studies for NP-induced toxicity as protection agents. The most studied phytocompounds in such studies are phenolic compounds and flavonoids such as quercetin, resveratrol, or curcumin (Table 1).

**Figure 2:**
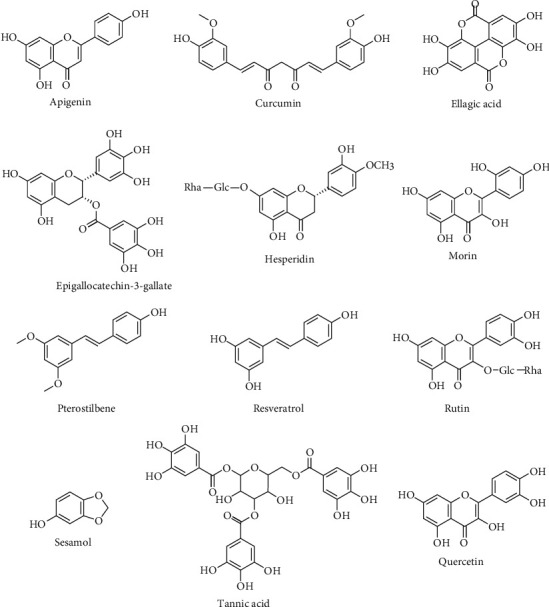
Plant phenolic compounds used in supplementation of nanoparticle-induced oxidative stress.

Quercetin (Figure 2) is one of the most studied dietary flavonoids present in many fruits, vegetables, and medicinal plants. Its antioxidant, anti-inflammatory, neuroprotective, chemopreventive, and cardioprotective properties are well documented. The bioactivity of quercetin is related to its high antioxidant and free radical scavenging activities [152, 158]. The antioxidant potential of quercetin in the suppression of NP-induced oxidative stress was examined using different models exposed to TiO_2_-NPs, AuNPs, CuO-NPs, and ZnO-NPs. Quercetin showed antioxidant protection of liver [152], kidney [153], and testicular [154] tissues in rats exposed to an overdose of TiO_2_-NPs. The concentration of TiO_2_-NPs used in these studies was in the range of 50 to 1000 mg/kg of body weight (b.w.) daily or once during the experiment, while the dose of quercetin was 75 or 200 mg/kg b.w. of tested animals daily. Quercetin displayed oxidative protection of liver, kidney, and testicular tissues lowering lipid peroxidation and improving antioxidant parameters of kidney and testicular tissues. Also, quercetin (75 mg/kg b.w. daily) significantly reduced the apoptotic index in kidneys [153] and testes [154], while mitigation of apoptotic marker caspase 3 and DNA fragmentation in liver tissue using 200 mg/kg b.w. daily for 21 days was observed [152]. The hepatoprotective activity of quercetin was also proven in AuNP-intoxicated rats reducing oxidative stress parameters in the liver [66, 149]. Quercetin was effective against CuO-NP-induced hepatotoxicity in rats. It is repented that coadministration of 150 *μ*g/kg b.w. quercetin daily for 3 weeks reduced significantly oxidative stress in liver tissue, serum levels of TNF-*α*, caspase-3 activity, and mRNA of Bax, while the significant elevation of the Bcl2 level was observed [150]. This research suggests that quercetin has the properties to inhibit some critical points of apoptosis. Similar antioxidant protection of quercetin against CuO-NP-induced hepatotoxicity in rats was observed in the study published by Arafa et al. [151], as well as against ZnO-NP-induced hepatotoxicity in rats [155].

Resveratrol (Figure 2), a stilbene, is also one of the commonly used plant antioxidants in the prevention of NP-induced toxicity. It can be found in different fruits, berries and medicinal and edible plants. Its antioxidant properties are the subject of many scientific publications, and results suggest that it possesses better antioxidant properties compared with vitamin E and C [143]. An *in vitro* study conducted by Ryu et al. [132] showed antioxidant effects of resveratrol on DNA damage induced by TiO2-NPs in lymphocytes. Resveratrol provoked significant decreases of ZnO-NP-induced prooxidant effects measured using DCFDA fluorescence intensity, mitochondrial damage, and apoptotic and necrotic effects in zebrafish embryos [144]. Pterostilbene, a stilbene chemically related to resveratrol, showed similar protection effects against AgNP-induced oxidative stress in zebrafish embryos [142]. The experiments with Nile tilapia fish showed that a fish diet supplemented with resveratrol enhances antioxidant protection parameters in fish gill, liver, and kidney disturbed by ZnO-NP application [140]. Resveratrol also displayed antioxidant protection of CuO-NP-induced oxidative stress in rats increasing total antioxidant capacity (TAC) and decreasing the total oxidant status (TOS) in serum [62]. Solaiman et al. [143] reported that resveratrol also has the ability to mitigate the increase of the serum MDA level induced by TiO2-NPs in an experiment with rats.

Curcumin (Figure 2), the main phenolic compound in spice turmeric, is reported as a natural antioxidant supplement against NiO-NPs, TiO_2_-NPs, and ZnO-NPs. Siddiqui et al. [130] reported that curcumin reduces ROS and lipid peroxidation levels, as well as increases the GSH level in NiO-NP-induced toxicity in human airway epithelial (HEp-2) and breast cancer (MCF-7) cells. Another *in vitro* study showed antioxidant protective effects of curcumin on DNA damage induced by TiO_2_-NPs in lymphocytes [132]. Curcumin also displayed a reduction in ROS generation measured using H_2_DCF-D dye on *Caenorhabditis elegans* worms exposed for 24 h to the LC_50_ concentration of TiO_2_-NPs and ZnO-NPs [131]. *In vivo* experiment on rats showed that coadministration of curcumin nanoparticles improved antioxidant parameters (TBARS, NO, GST, GPx, GSH, CAR, SOD, and total antioxidant capacity) and suppressed increased levels of tumor suppressor P53, TNF-*α*, and interleukin-6 in heart tissue of rats exposed to hydroxyapatite nanoparticles [133]. Apigenin is also one of the dietary flavonoids that showed hepato- and nephroprotective activities in animal models with NP-induced toxicity. Apigenin can protect the liver and kidneys against NiO-NP-induced toxicity [126] and kidneys against mesoporous silica nanoparticle- (MSN-) induced toxicity [127]. Apigenin in both mentioned studies significantly upregulated antioxidant parameters in analyzed tissues, while reducing the expression of TNF-*α* and IL-6 in the kidney of mice with MSN-induced toxicity. Similar protection activity of ellagic acid, as described for apigenin nephroprotection of MSN-induced toxicity in mice, was observed in a study conducted by Mohammed et al. [57] for IONP-induced nephrotoxicity in Wistar rats.

Among examined phenolic compounds in the protection of NP-induced oxidative stress, epigallocatechin-3-gallate was proved to be effective in the inhibition of oxidative stress developed with NiNPs in a mouse epidermal (JB6) cell line [135]. Epigallocatechin-3-gallate reduced intracellular ROS generation and cell apoptosis, significantly regulating the expression levels of AP-1 and NF-*κ*B and the MAPK signaling pathways disturbed by NiNP application. Nanoencapsulated silymarin and tannic acid showed potential to ameliorate hepatotoxicity in Nile tilapia fish [145] and hepatonephrotoxicity induced in rats [147] with AgNP-induced toxicity, respectively. These phenolic components modulated disturbed antioxidant parameters in the liver or renal tissues. Also, similar effects, with the regulation of antioxidant parameters GSH, CAT, GPx, SOD, and MDA in liver tissue, demonstrated coadministration of hesperidin parallel with ZnO-NP-induced oxidative stress in rats [138]. Flavonoids morin and rutin showed the same effects on testicular tissue of rats treated with TiO2-NPs [141]. Sesamol, a phenolic lignan from sesame oil, exhibited significant antioxidant protection effects of brain tissue in Al_2_O_3_-NP-treated rats [22].

Sulforaphane, an isothiocyanate compound found in cruciferous vegetables, also manifested antioxidant protection of CuO-NP-induced oxidative stress in BALB C3T cells [146], as well as DNA damage induced by TiO_2_-NPs in lymphocytes [132]. Glycyrrhizic acid, a natural sweetener isolated from the root of *Glycyrrhiza glabra*, showed the potential to reduce oxidative stress and the apoptotic process in the liver of rats treated with TiO_2_-NPs [137].

**Figure 3:**
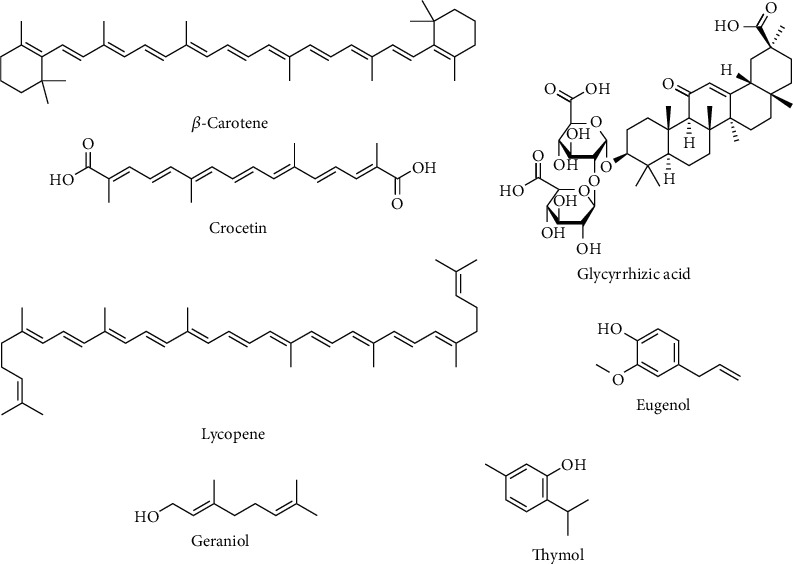
Plant terpenoids used in supplementation of nanoparticle-induced oxidative stress.

In addition to phenolic compounds, terpenoids and components of plant essential oils are often the subjects of NP-induced oxidative stress protection (Figure 3). *β*-Carotene showed potential to reduce the apoptotic index and increase CAT and GPx activities in the cerebral tissues of TiO_2_-NP-intoxicated rats [128]. A fish diet supplemented with lycopene manifested an increase of antioxidant protection parameters in Nile tilapia fish gill, liver, and kidney disturbed by ZnO-NP application [140]. Lycopene also enhances antioxidant protection and decreases cell apoptosis in testes of mice treated with TiO_2_-NPs [139]. CuO-NP-induced oxidative stress in the mouse hippocampal HT22 cell was effectively protected with coadministration of crocetin, a compound found in gardenia fruits and saffron [129]. It is found that crocetin increases the activity of antioxidant enzymes SOD and CAT, as well as GSH, SOD mRNA, CAT mRNA, and Bcl-2 mRNA levels in CuO-NP-intoxicated HT22 cell. The same study described the crocetin potential to reduce intracellular ROS and proapoptotic Bax mRNA levels in HT22 cells treated with CuO-NPs. The common constituents of essential oils eugenol, thymol, and geraniol are proven as antioxidant compounds in the prevention of oxidative stress in different organs induced by various NPs in experiments performed on rats [54, 134, 136, 148].

Available literature data about the use of phytochemicals as supplements in NP-induced oxidative stress showed the high potential of these compounds for application and development of new plant-based dietary supplements. The investigated phytochemicals are common constituents of aromatic, medicinal, and edible plants, and obtained results indicate a beneficial effect of the use of these plants or plant-based supplements in oxidative stress-related disorders. Also, these results are very useful for further research in this field suggesting that most of the known antioxidant phytocompounds have not been investigated yet as potential supplements in the treatment of oxidative stress induced by NPs.

## 4. Conclusion

Considering the many benefits of antioxidants, including vitamins, dietary supplements, plant products, and phytochemicals and their great potential in suppression of NP-induced oxidative stress, there is no doubt that the research that deals with the application of antioxidants in this field will continue with more attention in the forthcoming years. Different natural occurring antioxidants have been comprehensively reviewed in this paper. Although vitamins, plant extracts, and phytochemicals have shown great potential in NP-induced oxidative stress protection, understanding the mechanisms involved in the modulation of NP-induced oxidative stress of these natural products is still not fully understood. Hence, it seems that achieved results in this field present a good base for further research on protection mechanisms of NP-induced oxidative stress using naturally occurring antioxidants, as well as for more research including some earlier not tested plants and their bioactive compounds. The overall goals of future studies are dominantly focused to give an insight into new perspectives of NP usage with an imperative to decrease their toxicity using verified, safe, and validated antioxidant supplementary therapy that may be also beneficial for the living organism as a whole. Based on the analyzed results, one of the greatest potentials for further research is antioxidants of plant origin. A further strategy for the advancement of this area of research could be the related investigation for the use of new plant-based antioxidants as dietary supplements in the prevention or alleviation of oxidative stress symptoms associated with long-term exposure to NPs or their high concentration.

## Figures and Tables

**Table 1:** Plant extracts, essential oils, and phytocompounds used in antioxidant supplementation of NP-induced toxicity.

Extract/compound	Nanoparticles	Action	Reference
Extracts and essential oils			
Basil essential oil (nanoencapsulated)	IONPs	Hepatoprotection in rats	El-Nekeety et al. [111]
*Beta vulgaris* (beetroot) juice	AgNPs	Hepatoprotection in rats	Albrahim and Alonazi [5]
*Cinnamomum cassia* extract	TiO_2_-NPs	Hepatoprotection in rats	Shakeel et al. [107]
*Cinnamomum cassia* oil encapsulated with maltodextrin	TiO_2_-NPs	Increase antioxidant capacity in the liver and kidney and prevent genotoxicity and reproductive disturbances in male mice	Salman et al. [108]
*Eruca sativa* seeds	Hydroxyapatite NPs	Cardioprotection in rats	Alotaibi et al. [112]
*Filipendula ulmaria*	CaNPs	Reduce oxidative stress in brain tissue of rats as well as in liver, kidney, and testes tissues	Arsenijevic et al. [23] Scepanovic et al. [46]
*Foeniculum vulgare* (fennel) and *Pimpinella anisum* (anise) seeds	ZnO-NPs	Hepatoprotection in rats	Barakat [113]
*Gentiana asclepiadea*	AgNPs	*In vitro* DNA protection	Hudecová et al. [114, 115]
*Ginkgo biloba*	AgNPs	Hepatoprotection in rats	Abd El-Maksoud et al. [116]
		Improved neurotoxic side effects in rats	Lebda et al. [117]
Ginseng	SiO_2_-NPs	Reduce oxidative stress and apoptotic and inflammatory processes in rat lung	El-Sayed et al. [118]
Grape seed extract	TiO_2_-NPs	Hepatoprotection in rats	Mohammed and Safwat, [106]
Green tea extract	CuNPs	Hepatoprotection in rats	Ibrahim et al. [59]
*Moringa oleifera* leaf extract	TiO_2_-NPs	Nephroprotection in rats	Abdou et al. [109]
*Moringa oleifera* seed extract	TiO_2_-NPs	Cerebroprotective effect	Kandeil et al. [110]
	CuNPs	Enhance gill and liver oxidative damage in *Cyprinus carpio*	Noureen et al. [60]
*Pistacia lentiscus* essential oil	NiO-NPs	Decrease ROS generation in human lung epithelial (A549) cells	Mohamed et al. [119]
Pomegranate peel	AgNPs	Enhance liver and kidney damage, oxidative stress, and immunity biomarkers in Nile tilapia fish	Hamed et al. [120]
Pomegranate juice	CuO-NPs	Reduce oxidative stress manifestations in the brain through regulation of HO-1 and Nrf2 gens	Hassanen et al. [121]
		Antioxidant, anti-inflammatory, and antiapoptotic effects in the liver and kidney of rats	Hassanen et al. [122]
	AgNPs	Hepatoprotection in mice	Sallam et al. [123]
*Zataria multiflora* essential oil	IONPs	Hepatoprotection in rats	Attaran et al. [124]
Pumpkin seed oil	Al_2_O_3_-NPs	Antioxidant protection of rats' maternal and fetal hepatic and brain tissues	Hamdi et al. [125]
*Rosmarinus officinalis* extract	TiO_2_-NPs	Ameliorated plasma antioxidant markers in rets	Grissa et al. [105]
*Tinospora cordifolia* extract	TiO_2_-NPs	Enhance gill, liver and kidney oxidative damage and immunity biomarkers in Nile tilapia fish	Vineetha et al. [104]
*Zataria multiflora* essential oil	IONPs	Hepatoprotection in rats	Attaran et al. [124]
Phytocompounds			
Apigenin	NiO-NPs	Hepatorenal protection in rats	Ali et al. [126]
	Mesoporous silica nanoparticles (MSNs)	Nephroprotection in mice	Wang et al. [127]
*β*-Carotene	TiO_2_-NPs	Cerebroprotective effect	Abdel-kareem and Ayat Domouky [128]
Crocetin	CuO-NPs	Oxidative stress protection in HT22 cells	Niska et al. [129]
Curcumin	NiO-NPs	Reduce oxidative stress in human HEp-2 and MCF-7 cells	Siddiqui et al. [130]
	TiO_2_-NPs	Reduction of ROS generation in *Caenorhabditis elegans* worms	Sonane et al. [131]
		*In vitro* DNA protection in lymphocytes	Ryu et al. [132]
	ZnO-NPs	Reduction of ROS generation in *Caenorhabditis elegans* worms	Sonane et al. [131]
Curcumin nanoparticles	Hydroxyapatite nanoparticles	Cardioprotection in rats	Mosa et al. [133]
Ellagic acid	IONPs	Nephroprotection in rats	Mohammed et al. [57]
Eugenol	TiO_2_-NPs	Reduce oxidative stress in different organs of rats	Wani et al. [134]
Epigallocatechin-3-gallate	NiNPs	Reduce intracellular ROS generation and cell apoptosis in the JB6 cell line	Gu et al. [135]
Geraniol	ZnO-NPs	Neuroprotective effect in rats	Farokhcheh et al. [136]
Glycyrrhizic acid	TiO_2_-NPs	Hepatoprotection in rats	Orazizadeh et al. [137]
Hesperidin	ZnO-NPs	Hepatoprotection in rats	Ansar et al. [138]
Lycopene	TiO_2_-NPs	Reduce oxidative stress in testicular tissue of rats	Meng et al. [139]
	ZnO-NPs	Enhance gill, liver, and kidney oxidative damage in Nile tilapia fish	Abdel-Daim et al. [140]
Morin	TiO_2_-NPs	Reduce oxidative stress in testicular tissue of rats	Hussein et al. [141]
Pterostilbene	AgNPs	Prevent oxidative stress in zebrafish embryos	Chen et al. [142]
Resveratrol	CuO-NPs	Hepatorenal protection in rats	Khalid et al. [62]
	TiO_2_-NPs	Prevent testicular damage	Solaiman et al. [143]
		*In vitro* DNA protection in lymphocytes	Ryu et al. [132]
	ZnO-NPs	Prevent prooxidant mitochondrial damage and apoptotic and necrotic effects in zebrafish embryos	Giordo et al. [144]
		Enhance gill, liver, and kidney oxidative damage in Nile tilapia fish	Abdel-Daim et al. [140]
Rutin	TiO_2_-NPs	Reduce oxidative stress in testicular tissue of rats	Hussein et al. [141]
Sesamol	Al_2_O_3_-NPs	Neuroprotective effect in rats	Abou-Zeid et al. [22]
Silymarin	AgNPs	Hepatoprotection in Nile tilapia fish	Veisi et al. [145]
Sulforaphane	CuO-NPs	Reduce oxidative stress in BALB C3T cells	Akhtar et al. [146]
	TiO_2_-NPs	*In vitro* DNA protection in lymphocytes	Ryu et al. [132]
Tannic acid	AgNPs	Hepatorenal protection in rats	Mosa et al. [147]
Thymol	TiO_2_-NPs	Protection of testicular damage	Jafari et al. [54]
		Hepatoprotection in rats	Jafari et al. [148]
Quercetin	AuNPs	Hepatoprotection in rats	Abdelhalim et al. [66]
		Hepatoprotection in rats	Abdelhalim et al. [149]
	CuO-NPs	Reduce oxidative stress in the liver and antiapoptotic action in rats' liver	Abdelazeim et al. [150]
		Hepatoprotection in rats	Arafa et al. [151]
	TiO_2_-NPs	Hepatoprotection in rats	Fadda et al. [152]
		Nephroprotection in rats	Alidadi et al. [153]
		Protection of testicular damage	Khorsandi et al. [154]
	ZnO-NPs	Hepatoprotection in rats	Lotfy et al. [155]

## Data Availability

All data are available upon request.
